# Clinical records organized and optimized for clinical integration and clinical decision making

**DOI:** 10.5116/ijme.576a.fff4

**Published:** 2016-07-22

**Authors:** Allison A. Vanderbilt, Samay Jain, Sallie D. Mayer, Allison A. Gregory, Mark H. Ryan, Melissa K. Bradner, Reginald F. Baugh

**Affiliations:** 1 Family Medicine, College of Medicine and Life Sciences, University of Toledo, USA; 2 Division of Urologic Oncology, College of Medicine and Life Sciences, University of Toledo, Toledo, Ohio, USA; 3 Department of Pharmacy, Bon Secours,Virginia Health System, Midlothian, VA, USA; 4 Family and Community Health Nursing, School of Nursing, Virginia Commonwealth University, USA; 5 Department of Family Medicine and Population Health, School of Medicine, Virginia Commonwealth University, USA; 6 Department of Surgery, College of Medicine and Life Sciences, University of Toledo, Toledo, Ohio, USA

**Keywords:** Clinical records, clinical integration, clinical decision making

## Introduction

One of the most basic and cost effective skills a healthcare professional (HCP) can employ is correctly diagnosing (90%) a patient by taking an accurate medical history.^1^^,^^2^  A HCP will diagnosis and develop a treatment plan by requesting the following information from the patient: chief compliant, history of present illness, past medical and surgical history, medication history, family history, social history, and conducting a review of systems.  In an ideal situation, every patient would be able to recall all of this information immediately, accurately, systematically, and disclose it upon request.  However, such ideal conditions rarely exist in the field of health care, particularly amongst an aging population who are afflicted with greater numbers and complexities of comorbid health conditions. Also, economical pressures have forced many hospital systems and individual providers to refocus on the throughput of patients, which has led to less time spent with HCPs. As a result, patients have more health information and less time to convey it and usually end up disclosing: (a) chief complaint (or multiple complaints), (b) some medical history, (c) some family history and (d) a list of medications.  Moreover, as the patient moves through the health care process, he or she has subsequent opportunities to provide additional information which may be critical to the diagnosis/treatment of his/her ailment, but not available to every visited provider (i.e. pharmacist, physical therapist, etc.).  As a result, with the current system, the probability exists for the fragmentation of critical clinical information which can be unavailable for clinical decision making.

Historically, HCPs have used one of two major clinical reasoning paradigms for providing health care: decision making and problem solving.^3^^-^^6^ However, the successful implementation of these paradigms is contingent on complete and accurate documentation of clinical information and the availability of this information for medical decision making. This ideal is rarely met with the current medical record, as it is often proprietary and provider specific. Thus, HCPs are relegated to redundancy in history taking, which can result in errors, loss of efficiency, and ultimately, worse patient care and outcomes. 

One way to help improve the system is to leverage technology as a tool to help reduce inefficiency and inaccuracy. A systemized Electronic Health Record (EHR) program utilizing a common data repository with the ability to present different views of the clinical data with reminders of when new information is added, will allow for due diligence by HCPs to review all medical notes relevant to a patient. Thus, allowing for adequate time afforded by a health system and utilized by an HCP with each patient can reduce the likelihood that critical information is missed.  However, even with integration of technology into the patient care process, critical information can still be missed by individual HCPs. 

Options are available to help increase the fidelity of the medical record. It could be suggested that having one clinician return to ask the patient for the same information at various points would allow the information to be collected fully-the more points of contact by one HCP, the more complete the history will be. However, expecting a single HCP (especially a physician, who may not have much time to spend with a patient as compared to other HCPs) to return over and over again to the same question/topic may be impractical. Given that fact, the process of having multiple HCPs ask the same or similar questions at multiple points would improve the odds that information will be remembered/revealed, and this approach would be much more likely to be useful in a typical clinical setting. However, for this process to work, each member of the team must be familiar with the skills, training, and roles of the other team members, and have confidence in their ability to gather and interpret needed information. We propose that interprofessional education and collaboration in history taking will lead to the effective capturing and communication of patient information resulting in the best patient-centered outcomes.

### Definition interprofessional collaboration

Benefits of interprofessional education have been demonstrated in the literature. The Institute of Medicine’s seminal 2003 report entitled “Health Professions Education: A Bridge to Quality” called for health care students and working professionals to collaborate on interdisciplinary teams and engage in quality improvement.^7^ Regrettably, the United States ranks near the bottom among industrialized nations in every quality parameter measure, thus heightening the growing importance of quality care provided within interprofessional teams.^2^

Contentious battles among the health care disciplines have inhibited collaboration and teamwork.^8^ Kruse argues that health care providers: often lack respect for others, fail to recognize the value of a team-based approach and a shared vision, and demonstrate a deficiency in communication skills that are required to set goals and priorities aimed to improve health care efficiency and effectiveness. This health care attitude of working in silos exists because we have allowed and even nurtured competitive training programs rather than growing a rich environment grounded in interprofessionalism, teamwork, and collaboration.^8^ The focus of care needs to shift from the lens of any particular discipline to the lens of patient outcomes.  After all the patient comes to us for resolution of a health problem independent of who actually solves it.

Interprofessional collaboration is the foundation needed for health care providers to support patient needs^9^ and improve patient history taking.  We define interprofessional collaboration as two or more health care providers from different disciplines collaborating effectively together in order to improve health outcomes for their patients while working with patients, families, and communities to deliver the highest quality of care.^10^ Over the past decade, there has been a push toward interprofessional collaboration and teamwork.^6^ Interprofessional collaboration can bring forth the cooperation, communication, and teamwork necessary to provide a comprehensive health care plan to treat and care for the patient.^10^  The first step to achieving this goal is obtaining an accurate and complete patient history.

### Interprofessional collaboration: history taking

Interprofessional collaboration may assist with improving patient history taking because it brings a comprehensive team to treat the patient.  Through collective information sharing of the patient’s history and considering each HCP’s perspective on patient care, a comprehensive team of experts from various health care disciplines (e.g. physicians, pharmacists, nurses, and allied health providers)^7^ may obtain the most thorough understanding of the patient’s medical condition. 

In any healthcare setting patients have the tendency to share select pieces of their medical history with various health care professionals (team members).  A patient may disclose vital information that is necessary for the physician to know; however, it was told to the nurse.  Being a part of an interprofessional collaborative team, the nurse knows the information is critical and updates the EHR. Open, streamlined communication among the team members ensures that the patient history is always up-to-date and as complete and thorough as possible. The ability to communicate openly depends on an atmosphere of mutual trust and familiarity with the roles and responsibilities of each team member.^11^

Family members are another integral part of the patient history taking, in both the inpatient setting and the ambulatory setting where a spouse, parent or child might accompany a patient.  Some patients may be too sick to talk and their families or advocates may be the ones to disclose their medical history. The use of interprofessional healthcare teams allow for increased contact opportunities with family members that can lead to critical information gathering about the patient’s medical condition. An interprofessional team may also facilitate information sharing in cases where patients have been previously admitted to the same facility but in a different clinical setting.  Having an understanding of the temporal relationship and progress of a patient from one admission to another can be key to appropriate assessment and management. This is often the case for patients with complicated chronic disease conditions such as Lupus Erythematosus or Sickle Cell Disease. For example, a nurse or another HCP may recognize a patient from a previous visit and have information about the patient’s previous condition, progress, or specific response to a therapeutic measure that can be shared and communicated with other team members.  In the patient centered medical home model, a nurse phone call to the home for diabetes management for example might reveal information about an emergency room visit or hospitalization that is critical to the pharmaceutical and medical management.

Interprofessional collaborative teams can facilitate communication when shift changes, hand offs, the transfer of care to another area of the hospital or the transition from inpatient to the ambulatory setting. When a patient is transferred to another area of the hospital there may be onlyone team member that follows the patient, for example, the pharmacist.  Therefore, the pharmacist can update the new interprofessional collaborative team based on his or her understanding of what other team members may need to know.  He or she can provide an added perspective not robustly represented in the patient’s written history that may be important to the management of the patient.  “Collaborative care honors the diversity that is reflected in the individual expertise each profession brings to care delivery.”^12^ The understanding and realization of what information is relevant or needed by other disciplines to enhance patient management can be facilitated by interprofessional education and collaboration during the course of that HCP’s professional training.

Having interprofessional collaborative teams participate in patient history taking can provide other benefits to the patient and the team.  Some patients may feel more comfortable in disclosing their medical history to a healthcare provider based on their own or the provider’s gender, race, ethnicity, or age.  Despite the achieved cultural competency of the healthcare provider, an information gap may result beyond the provider’s control.  At times, language spoken by the patient may also pose a barrier to effective communication. An interprofessional team approach can facilitate information transfer from a specific care provider to the entire team, thus resulting in the best patient management plan. 

Finally, interprofessional collaboration and communication can lead to cost-saving measures.  By having a more thorough patient history, the HCP may be able to avoid a laboratory test, imaging study, or other follow-up procedures that could otherwise be unnecessary. Collaborative thinking and approach to the patient may also offer key insight to the team leader in implementing the most efficient and effective management care plan.  Potential trial and error situations can be avoided as well as loss of time that may be of utmost importance for patients in critical conditions.  This comprehensive team-based approach can bring extensive expertise that would otherwise not be present, thus, providing additional resources, health care services, and the potential for unexpected positive outcomes5 for not just the patient but the respective healthcare system and community.  Shared responsibility of patient care with team members in gathering the patient history lessens the burden of each HCP needing to gather all information independently which ultimately is less efficient due to frequent duplication of effort. 

### Educational Implications for HPCs

Achieving the goal of improving patient-centered care through a more collaborative patient history taking must begin with health professional education programs spearheading interprofessional education.  Programs can equip a workforce with new skills and with new ways of relating to patients and each other^8^ through teaching communication, effective skills, and patient history taking during clinical rotations or by using interprofessional teams when assessing patients in the outpatient setting. Within an interprofessional education competency framework, communication is considered a core aspect; however, health profession students often have little knowledge about or experience with interprofessional communication.^12^  

There are numerous demands for both retraining of the current health professions workforce and interprofessional learning approaches in order to prepare future health care practitioners.^7^ Therefore, incorporating interprofessional education into the daily routine of practice for all health professional students will potentially address the issue of unfamiliarity with other health professionals’ roles, and provide them with the foundation necessary to successfully participate on inpatient and outpatient teams. It is essential to note that being able to work effectively and efficiently as members of clinical teams is a key health professional student competency.^12^ Students must be exposed to this as frequently and consistently as possible in order to advance their readiness for residency or professional practice.

HCPs support the common curricular objectives to provide interprofessional collaborative opportunities to students during inpatient and outpatient clinical rotations, and expose students to a variety of patient encounters to assure that they experience patient history taking that is not asynchronous (e.g., what they experience from standardized patients) thus having a more realistic perspective of health care.  This can be achieved across all four years of medical school and integrated throughout the medical curriculum while simultaneously meeting the Liaison Committee Medical Education standard on interprofessional education^13^ and other health care professional programs, such as Pharmacy^14^  via rounding in teams.

Nurses are called on to practice to the full extent of their training and education through the 2010 IOM report, The Future of Nursing: Leading change, advancing health.^15^  This report highlighted the need for nursing education programs to collaborate with other health professional schools to provide interprofessional education in the clinical setting.  Interprofessional collaboration to improve quality and coordination of care is one of the main goals of the Campaign for Action, a nation-wide initiative to implement the recommendations included in the IOM Future of Nursing report.^16^

The Accreditation Council for Pharmacy Education’s 2016 includes a new standard that require schools of pharmacy to incorporate IPE into their curricula for accreditation. The standard highlights the need for pharmacy schools to create learning experiences that expose students to “patient-centered care in a variety of practice settings as a contributing member of an interprofessional team” through both didactic and experiential activities.  Students must specifically engage with other HCP students with the aim of improving interprofessional team effectiveness.”^17^

## Conclusions

It is essential to systematically obtain information via team members when collecting a patient history in order to have the most complete picture of the patient; thus allowing for the most accurate diagnosis and treatment of the patient. This can best be achieved in an inpatient and outpatient setting using interprofessional collaborative care and interprofessional education with students during their clerkships and rounding experiences to model and teach data collection, data sharing, and how to effectively and efficiently communicate with other team members.  This model has the potential to impact patient-centered care and the education of students’ while simultaneously improving patient history taking, which in turn may improve patient outcomes.

### Acknowledgements

The authors would like to thank Dr. Parham Jaberi for his initial discussion related to history taking and interprofessional education in Spring 2015.

### Conflicts of Interest

The authors declare that they have no conflict of interest.
